# Regeneration of the radial nerve cord in the sea cucumber *Holothuria glaberrima*

**DOI:** 10.1186/1471-213X-9-3

**Published:** 2009-01-06

**Authors:** José E San Miguel-Ruiz, Angel R Maldonado-Soto, José E García-Arrarás

**Affiliations:** 1 Department of Biology, University of Puerto Rico, Rio Piedras, Puerto Rico

## Abstract

**Background:**

Regeneration of neurons and fibers in the mammalian spinal cord has not been plausible, even though extensive studies have been made to understand the restrictive factors involved. New experimental models and strategies are necessary to determine how new nerve cells are generated and how fibers regrow and connect with their targets in adult animals. Non-vertebrate deuterostomes might provide some answers to these questions. Echinoderms, with their amazing regenerative capacities could serve as model systems; however, very few studies have been done to study the regeneration of their nervous system.

**Results:**

We have studied nerve cord regeneration in the echinoderm *Holothuria glaberrima*. These are sea cucumbers or holothurians members of the class Holothuroidea. One radial nerve cord, part of the echinoderm CNS, was completely transected using a scalpel blade. Animals were allowed to heal for up to four weeks (2, 6, 12, 20, and 28 days post-injury) before sacrificed. Tissues were sectioned in a cryostat and changes in the radial nerve cord were analyzed using classical dyes and immmuohistochemistry. In addition, the temporal and spatial distribution of cell proliferation and apoptosis was assayed using BrdU incorporation and the TUNEL assay, respectively.

We found that *H. glaberrima* can regenerate its radial nerve cord within a month following transection. The regenerated cord looks amazingly similar in overall morphology and cellular composition to the uninjured cord. The cellular events associated to radial cord regeneration include: (1) outgrowth of nerve fibers from the injured radial cord stumps, (2) intense cellular division in the cord stumps and in the regenerating radial nerve cords, (3) high levels of apoptosis in the RNC adjacent to the injury and within the regenerating cord and (4) an increase in the number of spherule-containing cells. These events are similar to those that occur in other body wall tissues during wound healing and during regeneration of the intestine.

**Conclusion:**

Our data indicate that holothurians are capable of rapid and complete regeneration of the main component of their CNS. Regeneration involves both the outgrowth of nerve fibers and the formation of neurons. Moreover, the cellular events employed during regeneration are similar to those involved in other regenerative processes, namely wound healing and intestinal regeneration. Thus, holothurians should be viewed as an alternative model where many of the questions regarding nervous system regeneration in deuterostomes could be answered.

## Background

Post-traumatic nervous regeneration is a rarely seen phenomenon in the central nervous system (CNS) of adult higher vertebrate animals. Organisms with CNS injuries can suffer the loss of motor activity and sensory perception and, as a result, develop limitations or disabilities. Some animal species, on the other hand, are capable of functional regeneration after an injury to their CNS. In these cases, severed neuronal fibers can find their targets through axonal path finding and neurogenesis can be activated. Efforts in different fields have generated a large amount of information as to what directs or limits the potential of neuronal cells to achieve proper functional regeneration. However, continued research and new approaches are still needed before we fully understand what governs the dynamics of injured nervous tissues and its regeneration.

One of the approaches that have been used to study nerve regeneration is the comparative analysis to determine the differences between those species that readily regenerate their CNS and those where regeneration is more limited [1-4]. The regeneration capabilities of the CNS have been said to decrease as we move higher in the phylogenetic tree [4,5]. Thus, relatively simple organisms such as Hydra and Planaria, can regenerate their neuronal network after amputation of a body part [6]. Similarly, studies with mollusks and annelids (including leeches) have shown that they are capable of regeneration after a crush-type injury to one of the CNS tracts [5]. However, the assumption that organisms in the lower phylogenetic scale are more able to regenerate their nervous system is not always true. This is highlighted by the fact that crustaceans, as well as two of the most studied invertebrate model animals, *C. elegans *and *Drosophila*, show limited CNS regeneration [5,7,8].

Among deuterosotomes, most of the studies on CNS regeneration have been done on the spinal cord of vertebrates. Here again, organisms can be grouped into good regenerators and poor regenerators. Among the former are lampreys, fishes, and urodele amphibians, the latter include most amphibians, reptiles, birds and mammals [4,9-11]. Some fish species have served as models to study axonal regeneration in the CNS of vertebrates, as these animals are capable of functional regeneration after a CNS injury [2,11,12]. Urodeles are among the most gifted to regenerate their CNS; following an injury they can regenerate a spinal cord with some functional recovery at any recovery time during their life cycle [3,13,14]. Other amphibians are also capable of regrowing spinal nerves after amputation of the tail, but only in an immature state [15,16]. Even though these models have provided detailed descriptions of the problems and limitations that neuronal tissues must overcome before functional regeneration, still a great deal of basic knowledge is missing to understand how this phenomenon could be regulated and neuronal regeneration enhanced.

CNS regeneration has been little studied in lower deuterostomes. In ascidians, the regeneration of the neural complex has been shown to occur after complete ablation [17,18], but no study of nerve transection or partial injury is available. There is no information on CNS regeneration in hemichordates or cephalochordates. In echinoderms, organisms with an amazing regenerative capacity, a few publications provide some information on CNS regeneration, mainly describing what occurs following arm amputation in asteroids and crinoids [19-22]. However, these experiments provide a brief description of nerve regeneration that is part of an overall description of how the new arm forms or focus on some specific processes such as cell proliferation during regeneration. Only recently has a study on CNS regeneration in echinoderms been done, but it is limited to transmission electron microscopy [23]. Our work confirms some of their findings and greatly expands the analysis of CNS regeneration in echinoderms.

In this study we use the sea cucumber *Holothuria glaberrima *to study nervous system regeneration. *H. glaberrima*, is an echinoderm of the class Holothuroidea, and as such, is an invertebrate deuterostome, placing this organism in close evolutionary relationship with vertebrates. Moreover, these organisms have been known for a long time to possess tremendous capabilities to functionally regenerate most of their organs and tissues in a very short time [24,25]. We have previously used this model system to study intestinal regeneration [26], the regeneration of the enteric nervous system [27] and the wound healing of the body wall [28].

**Figure 1:**
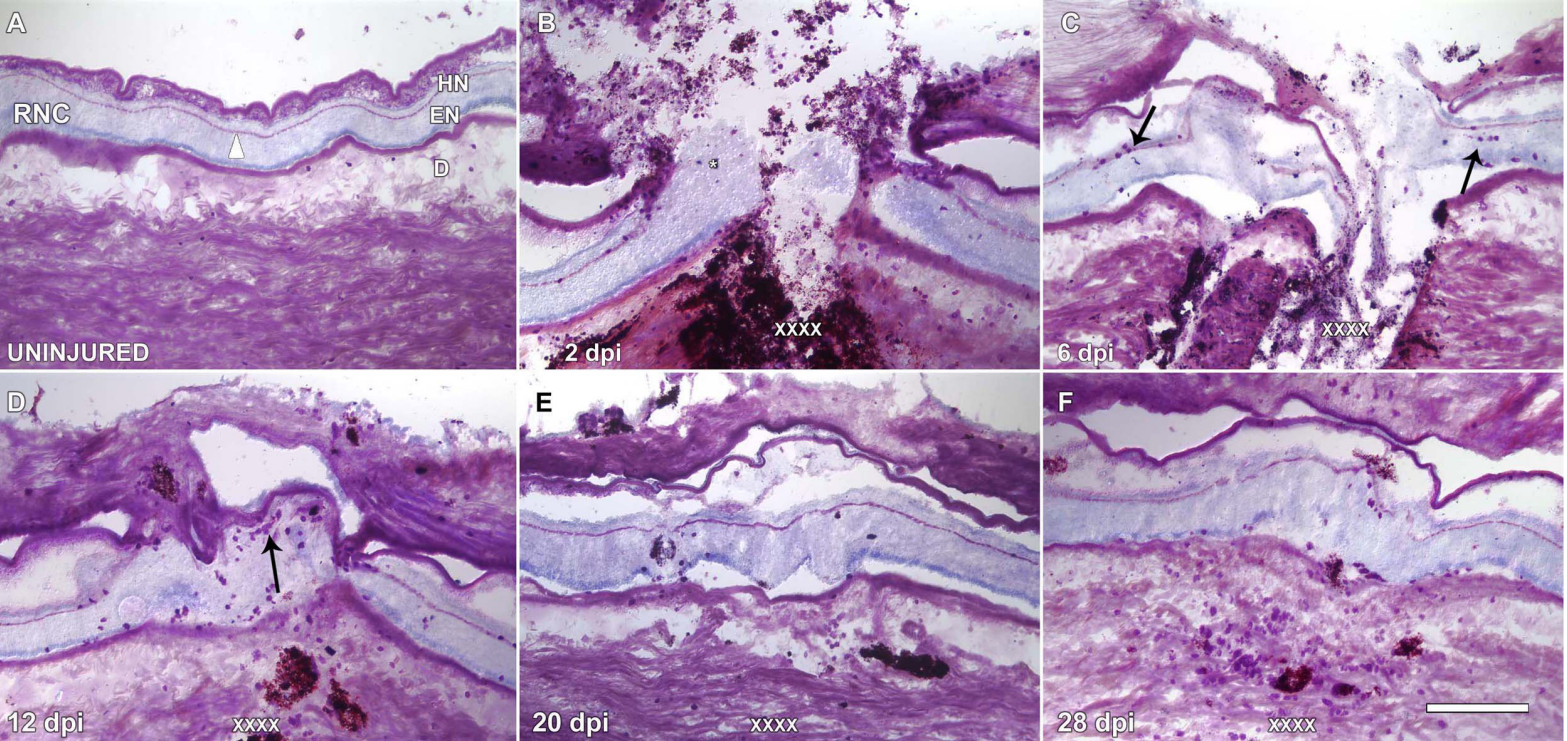
**Uninjured and regenerating stages of the radial nerve cord (RNC) of *H. glaberrima***. Longitudinal tissue sections of (A) uninjured and regenerating RNC at (B) 2, (C) 6, (D) 12, (E) 20 and (F) 28 days post injury (dpi) were stained with Toluidene Blue. (A) The uninjured RNC clearly shows the large ectoneural (EN) component separated from the thinner hyponeural (HN) component by a connective tissue band. (B) At 2 dpi the area is filled with debris and the open end of the RNC is visible (arrowhead). (C) By 6 dpi, the RNC stumps have organized into club-shaped structures. (D) By 12 dpi the injury gap has been filled with nervous tissue that now forms a continuous extension that joins the stumps. (E) By 20 dpi the RNC still appears slightly disorganized and no connective tissue band is observed. (F) At 28 dpi, the RNC has recovered much of the structure and organization found within non-injured RNC including the separation between the EN and HN components by a connective tissue band. D-dermis, EN-epineural component, HN-hyponeural component, RNC-radial nerve cord. X's denote the injury site; Asterisks show the presence of tattoo ink used to label the injury site; arrows signal morula cells within the RNC. Bar = 300 μm.

The holothurian CNS main component is an anterior nerve ring, at the base of the tentacles, from which 5 radial nerve cords (RNCs) exit and travel within the bodywall, spanning the length of the animal and ending blindly at the posterior end [24,29,30]. RNCs are subdivided into two components, the ectoneural (EN) or hyponeural (HN) band which are separated by a band of connective tissue (see Fig. 1A). In general terms the HN component is thought to be primarily motor, while the EN has both sensory and motor functions. RNCs are ganglionated nerves, meaning that they have neurons throughout their length. The neurons can be found in the periphery of both HN and EN components with the central portion, or neuropile, made up of nerve fibers. Innervation of organs occurs by peripheral nerves that emerge from the RNCs. We have developed a technique that allows for an accurate injury to be made to the RNC and are now able to study the regenerative potential of the holothurian CNS. We report here that following transection, re-construction of the nerve stumps occurs by 12 dpi, as a result of axonal growth; afterwards the RNC goes through a remodeling phase to achieve its original morphology. Other events associated with tissue development and regeneration were also observed, including cell division, migration and apoptosis. With this work we set the foundations for further studies on nervous regeneration by showing that this animal model carries out the two most important processes for CNS regeneration, explicitly neurogenesis and axonal pathfinding. More importantly, these occur as a natural response to the injury, allowing for future characterization of a permissive environment for possible therapy-related studies.

## Methods

### Animals

*Holothuria glaberrima *specimens, 10–16 cm long, were collected from the northeastern rocky shores of Puerto Rico and kept in an aquarium at 22–24°C with circulating seawater and constant oxygenation. All experiments conform to the regulations on animal research at the University of Puerto Rico.

### Surgical procedures

The technique used to injure the RNC has been described elsewhere [28]. Briefly, evisceration was induced by intracoelomic injection of 0.35 M KCl (3–5 ml). One hour following evisceration, animals were anesthetized by placing them for 10 min in 0.1% 1,1,1-Trichloro-2-methyl-2-propanol hydrate (chlorobutanol) diluted in seawater. Severing of the RNC was done by pushing the dorsal bodywall through the cloaca with a blunt rod, thus exposing the coelomic surface of the bodywall. A transection-type injury was made perpendicularly to the nerve using a scalpel, after which animals were reverted to their normal orientation and returned to their aquarium. By transecting the radial nerve from the inside we could obtain a reliable injury to the RNC with minimal damage to the surrounding tissues. Control animals were taken through the same protocol, except that no transection was made.

### Tissue harvesting and Histology

During the regeneration period, animals were sacrificed at different days post-injury (2, 6, 12, 20, and 28 dpi). Animals were anesthetized with chlorobutanol as before, slit open and pinned to a dissecting tray. Initially, they were directly fixed within the tray with 4% paraformaldehyde for 30 min and then the nerve-muscle complex, along with the bodywall, was excised. The tissue block was refixed overnight, washed in PBS and left in 30% sucrose until used.

Tissue blocks were embedded in OCT (Tissue Tek OCT; Miles Inc.), frozen at -35°C and longitudinally sectioned (20 μm) in a cryostat (Leica CM 1900). Sections were mounted on poly-L-lysine coated slides, left to dry for 1 hr at room temperature and stored in a dry chamber until needed. Since the RNCs are localized within the body wall, the tissue sections contained the regenerating RNC, the proximal regions that were 0.5 mm from the injury site, and the distal regions that were approximately 5 mm away.

### Histological analyses

#### Chemical Dyes – Toluidine blue

To obtain a comprehensive view of the temporal modifications that the RNCs go through after an injury, longitudinal tissue sections were stained with toluidine blue, as described by Presnell and Schreibman [31]. In brief, sections were rinsed in PBS for 2 min, and then immersed in dye solution for 2 min. They were then rinsed with tap water, mounted in PBS-buffered glycerol and viewed under the microscope. Occasionally, staining was also done on tissue sections previously processed for immunohistochemistry (see below).

#### Morphometric analysis

To evaluate changes in the RNCs following injury, tissue sections stained with toluidine blue were studied under light microscopy and the shortest distance between nerve stumps of injured animals was recorded. At least ten tissue sections were measured per animal and three to five animals were used for each stage. Sections were examined and photographed using a Nikon E600 microscope. Images were collected digitally with an RT Color Spot camera (Diagnostic Inst. Inc.) with 1520 × 1080 pixel resolution, and analyzed using the MetaVue software (version 6.0; Universal Imaging, Inc.) and Image J (version 1.37;NIH, USA).

#### Immunohistochemistry

Protocols for the immunohistochemical procedures performed for this study have been described elsewhere [28]. Tissue sections were permeabilized with 0.5% Triton X-100 in PBS, blocked with goat serum (1:50), and incubated at room temp overnight with the corresponding primary antibody: α-Bromodeoxyuridine (BrdU, 1:5, Amersham), Sph2 supernatant [32], α-GFSKLYFamide (α-GFS, 1:1000, [33]), α-Galanin (1:500, [34]), α-Nurr1 (1:1000, Sigma) or RN1 (1:50,000, [35]) in a humid chamber. The following day, sections were rinsed in PBS and treated with the FITC-labeled goat-anti-mouse secondary antibody (1:50, BioSource Int.). When double labeling was performed, the Cy3-labeled goat-anti-rabbit antibody (1:2,000) was used along with the FITC labeled antibody. Sections were rinsed again in PBS and mounted in buffered glycerol. Double or triple labeling with Hoechst nuclear dye (Sigma) was done by immersing slides in 1 μM Hoescht for 15 min after the secondary antibody [28]. Sections were examined and photographed using the Nikon E600 microscope equipped with FITC, R/DII and DAPI filters. Images were recorded using the MetaVue software as mentioned previously.

#### Cell counts

Various cellular populations were labeled and quantified. These include (1) the overall number of cells within the RNC using Hoescht nuclear dye, (2) spherulocytes, cells that contained a large number of spherules within their cytoplasm and were labeled with monoclonal antibody Sph2, (see [32]), (3) morulas, cells that also contain vesicles or spherules and that are strongly stained with Toluidine blue (see [32]), (4) neuropeptide-containing neurons labeled with anti-GFS and (5) a neuronal population labeled with an antibody against the transcription factor NURR1. The number of cells was quantified by counting labeled cells in a 280 mm^2 ^area of the longitudinal RNC (this is the equivalent of the microscope field of view-FOW using the 40× objective). In order to be counted, cells had to be within the nerve or in close apposition with the somas in the nerve periphery, or with the fibers that had exited the nerve stumps. Cell counts were done in two fields of view chosen at random for each corresponding area in every animal and in at least five animals per regeneration stage.

#### TUNEL assay

The TUNEL method was used to label apoptotic cells within the RNC. For this purpose we used the TdT-FragEl DNA Fragmentation Detection kit (Calbiochem QIA33) and followed the manufacturer specifications with only minor modifications. Tissue sections were washed for 3 min in PBS to remove the embedding medium; afterwards they were refixed in 4% formaldehyde (diluted in 1× PBS) and washed in TBS for 15 min. Tissue sections were permeabilized with 50 μl proteinase K (5 μg/ml) for 15 min and then washed in TBS. Prior to labeling apoptotic cells, tissue sections were bathed with 1× TdT Equilibration Buffer (80 μl per section) for 30 min. For the labeling reaction, 60 μl of the mixture (57 μl Labeling Reaction Mix: 3 μl TdT enzyme) were applied to each section and incubated for 1 hr at room temperature. Slides were then washed 2× for 2 min in TBS, counterstained with the nuclear dye Hoescht for 10 min, washed for 5 min in TBS, and mounted with phosphate buffered glycerol.

#### Proliferation Assays

##### Pulse-chase experiments

Incorporation of BrdU into cell nuclei was used to study the spatio-temporal distribution of proliferating cells in regenerating animals. Animals were injected intracoelomically at 2, 6, 12, 20 and 28 dpi with 100 μl of a 1 mg/ml BrdU solution (approximately 0.01 mg/g wet weight) and sacrificed 24 hrs after. Tissues were fixed and treated as described above for immunohistochemistry. Cells undergoing replication were identified with an antibody against BrdU (Amersham). Prior to the incubation with the antibody, tissue sections were bathed with 0.05 N HCl for 1 h. This was done to improve the accessibility of the antibody to the BrdU epitope, followed by an additional 15 min PBS wash. Cell division index was obtained by dividing the number of BrdU-positive cells against the total number of Hoescht-labeled nuclei within a longitudinal section of the RNC in the microscope field of view. At least two measurements of corresponding areas chosen at random from each of at least five different animals were used for each stage.

##### Cellular birth-dating

To determine if dividing cells were giving rise to the neurons in the regenerating RNC, we injected animals with BrdU every other day for a period of 6 days (4 BrdU injections per animal in each group). There were a total of 6 groups: 0–6, 8–14, 16–22, 24–30, 32–38, and 40–46 dpi; all animals were sacrificed at 62 dpi. The rationale behind the multiple and consecutive doses of BrdU stems from the assumption that new neurons do not differentiate immediately after the division, but that a dividing neuronal precursor will not necessarily express the neuronal markers after the last division. Multiple injections were thought to be necessary to assure that at least some cells were caught at the time of division. Sections of RNCs from the various groups were double labeled against BrdU and markers for different subpopulations (GFS, Galanin, Nurr1) to establish the date of birth of the neurons that were repopulating the new RNC tissue.

### Statistical Analyses

Student's t test and ANOVA's were used for statistical analyses. P values of < 0.05 were deemed to be significant.

## Results

### Overview of nerve regeneration

To assess the morphological changes that the RNC underwent during the regeneration period, longitudinal sections of body wall were stained with Toluidine blue and studied under light microscopy (Fig. 1). The first stage (2 dpi) was characterized by an injured RNC with fan-shaped stumps (Fig. 1B). Remarkably, the pre-existing CT band in the stumps were practically unaffected; if anything, it lost a bit of cohesiveness at its most proximal end, adjacent to the injury. By 6 dpi, small bundles of fibers had extended from both nerve stumps, giving the structure an overall needle-like shape laying on the substratum provided by the body wall and pointing towards the opposing stump (Fig. 1C). At this stage, the origin of nerve bundles extending beyond the stump seemed to be, mostly, the result of an outgrowth in the nerve EN component, which lies closer to the body wall. Still, the lack of a substratum in the injury gap appeared to be the main limitation for scouting bundles. Support for this comes from observations of fiber bundles, which in the absence of a direct intervening matrix between the stumps, were found close to the trough of the injury gap. Nerve re-contact occurred by 12 dpi, concurrent with the appearance of a provisional matrix that filled the injury gap (Fig. 1D). The regenerated RNC spanned the injury gap but remained disorganized with empty spaces that could be seen. This was clearly observed by using the differential interference contrast (DIC) filter in the microscope. Although the nerve had reconnected by 12 dpi, the CT band that normally separates the HN and EN was not present. The RNC structure was much improved by 20 dpi (Fig. 1E). At this stage the number and localization of nuclei, both in the HN and EN, was similar to that of an uninjured nerve. The CT band separating the HN and EN was already perceptible at this stage in some, but not all animals, as traces of thin filaments along the length of the nerve. Approximately one month after the injury (28 dpi), the regenerated nerve had a normal architecture (Fig. 1F). The nuclear arrangement and density of fibers was now similar to that of a normal uninjured nerve and the CT band was already present and compact, although in some areas it still seemed diffuse.

### Regeneration of fiber subpopulations

To obtain an in-depth view of the events that occur in the regenerating RNC, we studied the responses of different fiber subpopulations to the injury. Using immunohistochemistry we were able to correlate the gross appearance of the RNC, studied in light microscopy, with the growth of particular fiber populations across the injury site of the regenerated RNC.

#### RN1

**Figure 2:**
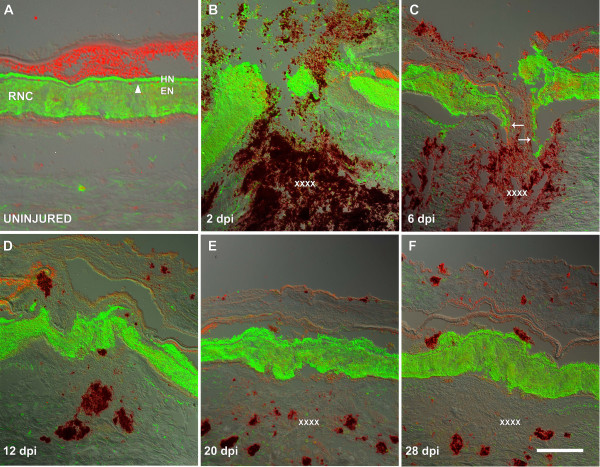
**RN1 labeling of the regenerating radial nerve cord**. Longitudinal tissue sections of (A) uninjured and regenerating radial nerve cords at (B) 2, (C) 6, (D) 12, (E) 20 and (F) 28 days post injury (dpi) were labeled with the monoclonal antibody RN1. (A) Labeling in the uninjured RNC is highly specific to the nervous components, both ectoneural (EN) and hyponeural (HN) component while the connective tissue band remains unlabeled (arrowhead). (B) At 2 dpi nerve fiber debris is visible around the injury site and the nerve stumps can be seen to be highly disorganized. (C) By 6 dpi, the RNC stumps have organized into club-shaped structures from which some nerve fibers can be observed to be extending (arrows). (D) By 12 dpi the injury gap has been filled with nervous tissue that now forms a continuous extension that joins the stumps. (E) By 20 dpi the RNC still appears slightly disorganized and the CT band is not present along the entire length of the regenerated RNC. (F) At 28 dpi, the RNC has recovered much of the structure and organization found within non-injured RNC including the connective tissue that separates the EN and HN components. EN-epineural component, HN-hyponeural component, RNC-radial nerve cord. X's denote the injury site; Bar = 300 μm.

This is a monoclonal antibody raised in our lab against *H. glaberrima *RNC tissue [35] that provides very specific labeling of what appears to be a large fiber population of the holothurian nervous system (Fig. 2A). RN1 does not label the CT band that separates the HN and EN component, thus, this band could be detected as a non-labeled distinct strip of tissue embedded within the high density of RN1 immunoreactive fibers. As a result, this antibody served as a two-edged marker, to measure progress during the process of nerve regeneration, namely the tracking of fibers as they elongate, and the appearance of the CT band as evidence of tissue organization into HN and EN components.

Most of the data collected with this antibody confirm the observations made using the Toluidene blue dye; however, some interesting differences were found. The fan-shaped morphology of the nerve stumps at the injury site observed at 2 dpi was mostly the result of a swelling (enlargement) in the EN component, while the HN component was not severely affected (Fig. 2B). At this stage, the HN component proximal to the injury appeared to retract in relation to the EN. At 6 dpi, the stumps acquired a club-shaped appearance and RN1-labeled fiber bundles could be observed protruding toward the opposite stump (Fig. 2C). Unfortunately, because of the high density of labeling, individual nerve fibers inside these pioneering bundles were hard to identify. However, the RN1 fiber bundles were always observed to be in direct contact with the substratum of the body wall and continuous with the injured stump. At this stage we observed the first difference in the rate of regeneration between the EN and HN components; the EN component provided the first scouting bundles that traverse the substratum towards the injury site, while RN1-labeled fibers of the HN appeared to be in a latent state. At 12 dpi, RN1 fibers from opposing nerve stumps came together, forming the newly regenerated RNC (Fig. 2D). At this stage, although RN1 fibers filled the area between the stumps, the separation of the RNC into its respective components had not occurred yet. During this stage RN1 labeled side branches could be observed originating from the same nerve bundles that form the connection between the stumps. These branches might be some that had gone astray into other injured tissues or they might be precursors to peripheral nerves. By 20 dpi the new RNC showed the first hint of connective tissue separation between the HN and EN components, albeit, rudimentary and irregular at times (Fig. 2E). The EN component was now structurally comparable to that of an uninjured RNC, as its wandering bundles of fibers were no longer present or at least apparent. Additionally, the regenerated RNC had a more cylindrical shape in accordance with the rest of the nerve; nonetheless, the staining intensity was now less than in uninjured areas. In spite of all these improvements in the EN component, the HN in most animals was still underdeveloped compared to uninjured counterparts in the same section. The new RNC was restored by 28 dpi with an HN and an EN component clearly separated from each other by the CT band between them (Fig. 2F).

#### α-GFS

α-GFS is a polyclonal antibody made in our lab against the neuropeptide GFSKLYF-amide that recognizes a subpopulation of neuronal fibers and somas in both HN and EN components of the RNC of *H. glaberrima *[33].

**Figure 3:**
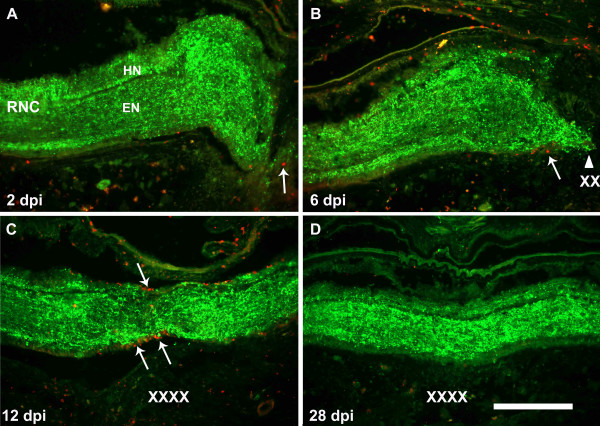
**Double labeling of regenerating radial nerve cords for the fibers expressing the neuropeptide GFS and proliferating cells with BrdU**. Longitudinal tissue sections of regenerating RNC at (A) 2, (B) 6, (C) 12, and (E) 28 days post injury (dpi) were labeled with anti-GFS (green) and anti-BrdU (red). Animals that had undergone a transection of their radial nerve were injected with BrdU 24 hrs prior to sacrifice. (B) GFS expressing fibers can be seen to elongate out of the nerve stump by 6 dpi (arrow). (D) Restoration of GFS-expressing fibers in the radial nerve cord is complete by 28 dpi. In terms of cell division, little cell division is observed at (A) 2 or (D) 28 dpi. Actively dividing cells were mainly observed in the periphery of the RNC where most of the nuclei are present (arrows). Some cell division is also observed among cells in the neuropile. Cell division begins around (B) 6 dpi and peaks around (C) 12 dpi in both hyponeural and ectoneural components (arrows). EN-epineural component, HN-hyponeural component, RNC-radial nerve cord. X's denote the injury site; arrows signal some of the BrdU-labeled cells within the RNC. Bar = 300 μm.

At 2 dpi, immunolabeling with GFS clearly showed the fan-shaped morphology of the cut RNC and the fraying of the nerve fibers close to the injury (Fig. 3A). The reduction in fiber density close to the injury was accompanied by an increase in labeling intensity close to the nerve stumps, which could be attributed to the appearance of α-GFS-labeled varicosities along the fibers. At 6 dpi, GFS-immunoreactive fibers had sprouted from the nerve stumps and the overall structure was clearly pointing towards the opposite stump (Fig. 1B). It is important to note that that the previously observed varicosities were also present in these migrating fibers. Similar to what was observed for RN1, fibers expressing the GFS neuropeptide also reconnected by 12 dpi (Fig. 3C). The labeling with GFS showed that immunoreactive fibers in the HN followed those of the EN; instead of using the body wall as a substrate. Even when the path taken by some fibers might be twisted and indirect, the overall axis of the fibers inside the regenerated EN was parallel to the long axis of the RNC. By 20 dpi both components were present in the regenerated RNC. No major differences were observed a month after transection (28 dpi) between regenerated RNC segment and its uninjured counterpart (Fig. 3D).

#### α-Galanin

**Figure 4:**
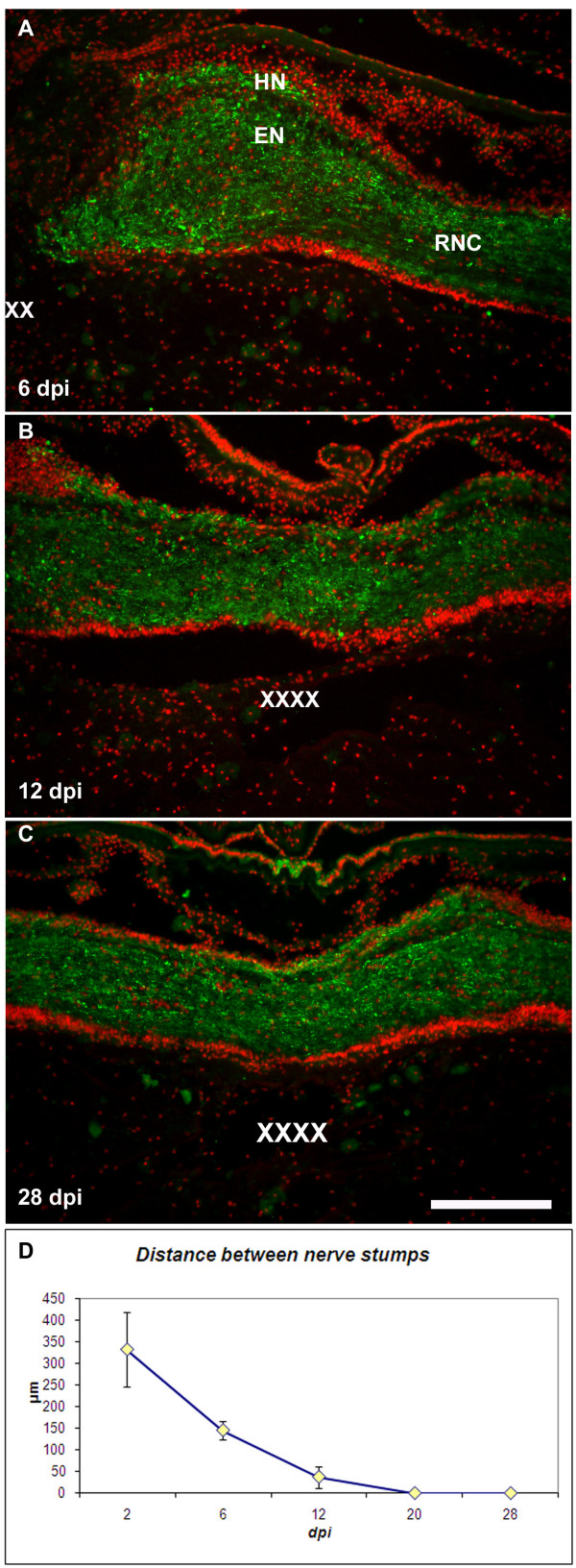
**Galanin-like immunoreactivity in regenerating radial nerves**. Longitudinal tissue sections of regenerating radial nerve cords at (A) 6, (B) 12 and (C) 28 days post injury (dpi) were labeled with anti-galanin (green) and counterstained with Hoescht (red) to label cell nuclei. (A) Galanin immunoreactivity disappears from the injury edge of the cord (asterisk) and the proximal stump acquires a punctuated labeling at 6 dpi. (B) At 12 dpi, the immunoreactive fibers have crossed the injury gap and labeling appears more homogeneous. (C) At 28 dpi labeling is similar to that in the uninjured cord (not shown). EN-ectoneural component, HN-hyponeural component, RNC-radial nerve cord. X's denote the injury site. Bar = 300 μm.

In the RNC this antibody labels a relatively small population of fibers [34]. At 2 dpi, galanin immunoreactive fibers underwent a change in their morphology forming round varicosities with very intense labeling. This change was more conspicuous closer to the injury site, where the nerve fibers have became frayed; they were present in the HN and EN component of the RNC. At 6 dpi, varicosities were also found in the needle-shaped bundle of fibers exiting the nerve stump (Fig. 4A). Once the RNC reconnected at 12 dpi, the area of the injury could be easily identified by the presence of these large varicosities. At 20 dpi, galanin-positive fibers were present in both components of the RNC, although with a higher density in the EN (Fig. 4B). Galanin-positive fibers passing between these two components were also apparent at multiple points along the regenerated RNC, as is usual in an uninjured nerve. At 28 dpi, the large varicosities had disappeared and α-galanin produced a labeling that was virtually undistinguishable from that of a normal RNC.

In summary, all neuronal markers used showed that the injury gap that separates the two RNC stumps is slowly filled by a growth of fibers that originate from the stump ends in the first two weeks of regeneration (Fig. 4C). This process is complete by 20 dpi and a RNC with high similarity to the uninjured RNC is in place by 28 dpi.

### Cell division

#### Pulse chase

**Figure 5:**
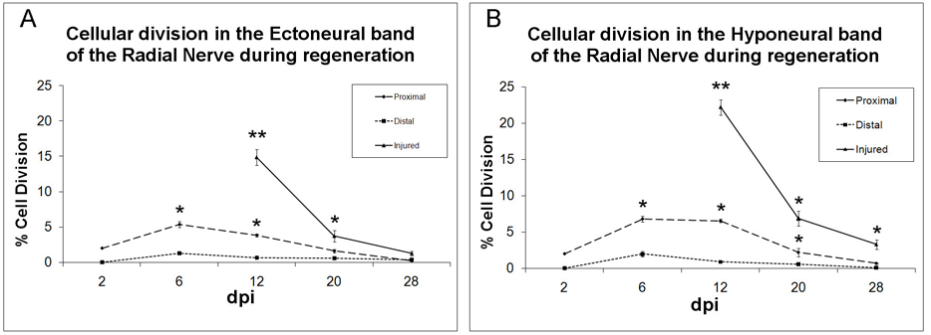
**Patterns of cell proliferation in the radial nerve cord**. The percentage of BrdU-labeled cells compared to Hoescht labeled nuclei in the (A) ectoneural and (B) hyponeural component of the radial nerve cord are shown. Measurements were done at the injury site and compared with sites proximal and distal to the injury. Animals were injected with BrdU 24 hrs before sacrificed. Quantification of cell division at the injury site shows a peak in cell proliferation at 12 dpi both in the hyponeural and ectoneural components. A smaller increase in cell division is also observed in the nerve stumps proximal to the injury site, but not so distal to the injury. Regenerated tissues in the injured area do not appear until 12-dpe. Each point represents the mean ± S.E. of at least five animals. When compared to distal, significant differences are observed at various stages in the proximal and injured areas *p < .05, **p < .01.

RNCs are ganglionated nerves where the neuronal soma and supporting cells can be mainly found in the periphery. To study the possibility that cell division is occurring in the RNC as a result of the injury, animals were injected with the thymidine analog, BrdU, and then checked for its incorporation into the nuclei. Initially, at 2 dpi few cells within the proximal stumps, both in the HN and EC bands, of the injured nerves were undergoing cell division; no cell division was seen distal to the injury site (Fig. 5). At 6 dpi, the highest numbers of dividing cells were seen in the proximal region, with about 6% of cells (6.81 ± 0.42 in HN and 5.36 ± 0.4 in EN) undergoing cell division. Cell division distal to the injury site never exceeded 2%. By 12 dpi, cell division in the proximal stumps remained comparatively similar (6.54 ± 0.23 in HN and 383 ± 0.21 in EN) to the previous stage. However, an impressive surge in cell division occurred in the regenerating RNC that is forming across the injury site, where close to 20% of the cells (22.2 ± 1.06 in HN and 14.9 ± 1.15 in EN) were undergoing cell division. Subsequently, at 20 dpi, a sharp drop in cell division was observed in the newly formed RNC. Proximal to the injured nerve, cell division did not exceed 2%. At the last stage studied (28 dpi), cell division was mainly restricted to the HN of the regenerated RNC, where cell division was 3.28 ± 0.7% in HN and 1.26 ± 0.3 in EN, everywhere else, cell division was less than 1%.

#### Cell regeneration and birth dating

**Figure 6:**
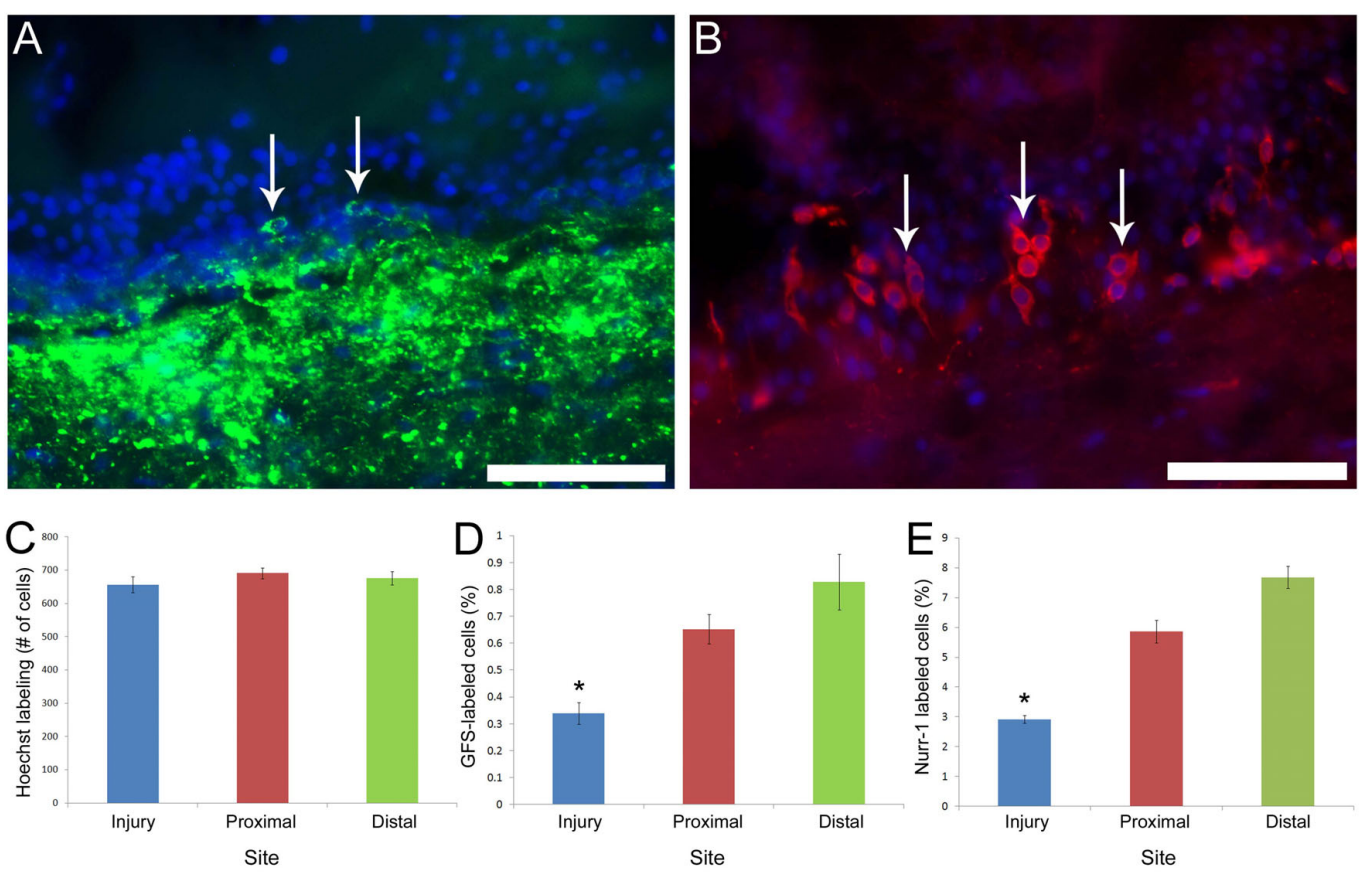
**Neuronal labeling in the regenerating radial nerve cord**. Neurons (arrows) within the regenerated radial nerves were observed 62 dpi using (A) anti-GFS and (B) anti-Nurr1 markers. (C) The total number of cells within the regenerated radial nerve is not significantly different from the number of cells at proximal or distal sites. In contrast, quantification of the percent of neurons at the three sites showed that the percentage of neurons at the injury site was observed to remain significantly lower than at the other two sites. Each point represents the mean ± S.E. of at least three animals. *p < .05, **p < .01. Scale bars = 50 μm.

The next step was to determine whether the neurons in the regenerated RNC were originating from dividing precursors. For this we used two neuronal markers that label independent neuronal populations in the RNC, anti-GFS [33] and anti-Nurr1 (unp. obs.). These markers labeled neuronal cell bodies in both the EN and HN components of the RNC (Fig. 6A &6B). Initially, experiments were made by injecting animals with BrdU at different time periods and sacrificing animals at 30 dpi. However, these animals showed few if any GFS or Nurr1 neurons in their regenerated RNC. Thus, we decided to extend the experiment until day 62 dpi. For this, animals were separated into 6 groups and each group received BrdU injections during a particular 6-day period. All animals were sacrificed at 62 dpi. The rationale for this experiment was that only those cells that stop proliferation close to the last BrdU injections would retain the BrdU label until 62 dpi. Cells that kept proliferating would dilute out the BrdU and not be labeled. Thus, we would expect that dividing neuroblasts would stop dividing and then differentiate, so that most of the labeled cells that had stopped dividing within the RNC would be neurons.

Sections of the regenerated RNC were obtained and the cells in an area of equivalent length of RNC were determined on the microscope field of view (40×) were counted at 62 dpi. Results showed that the total number of cells (as measured by counting Hoescht-labeled nuclei) within the regenerated RNC did not differ from that in the proximal and distal RNC, suggesting that cell regeneration had occurred after 2 months (Fig. 6C). At this time, neurons expressing GFS or Nurr1 were also found within the regenerated RNC, albeit, in smaller numbers when compared to those within the proximal and distal RNC (Fig. 6D &6E)

**Figure 7:**
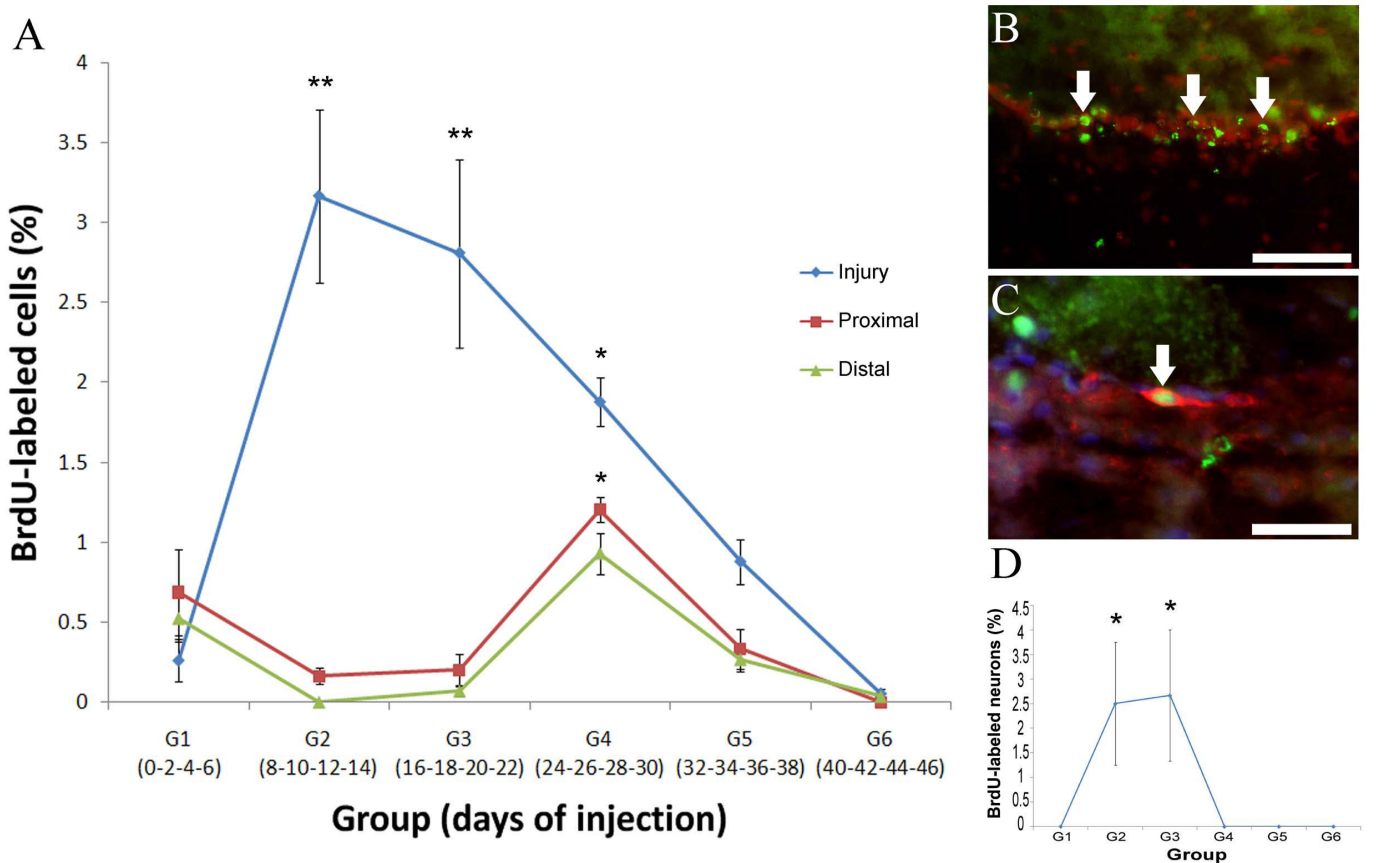
**Cellular birth dating in the regenerating radial nerve cord**. (A) Quantification of the percent of BrdU-labeled nuclei for each group of regenerating animals that were injected with BrdU at different time points and sacrificed at 62 dpi. (B) BrdU-labeled nuclei (green, arrows) among Hoechst-labeled nuclei (red) in the radial nerve cord at the injury site of an animal injected during the second week of regeneration. (C) Triple labeling showing Nurr1-expressing cell (red, arrow) with BrdU-labeled nuclei (green) in the hyponeural compartment at the injury site. Blue labeling is Hoechst dye. (D) Quantification of the percent of Nurr1 labeled neurons that were BrdU-labeled for each group at 62 dpi. The only neurons labeled with BrdU at 62 dpi were from the animals injected during the second and third weeks of regeneration (groups 2 & 3). Each point represents the mean ± S.E. of at least three animals. When compared to distal, significant differences are observed at various stages in the injured area. Similarly, significant cell division occurs in the proximal and distal area at G4. *p < .05, **p < .01. Scale bars = (A) 50 μm, (B) 25 μm.

Birthdating studies showed a peak during the second and third week of regeneration (groups 2 & 3, 8–22 dpi) when close to 3% of the cells (group 2 – 3.16 ± 0.54%, group 3 – 2.81 ± 0.59%) stopped dividing, and were still BrdU labeled at 62 dpi (Fig. 7A &7B). This would suggest that it is during this time period that most neurons have stopped dividing and started differentiation. To further verify that some of the dividing cells were neurons, we used double labeling with the neuronal markers GFS and NURR1. The results were somewhat unexpected. None of the cells that expressed GFS were double labeled with BrdU in any of the injected groups, even when over 300 GFS neurons were observed. In contrast, some cells labeled with Nurr1 were also labeled with BrdU (Fig. 7C). These cells were present only in the HN component of animals injected with BrdU during 8–22 dpi (which corresponds to the peak in cells that stopped proliferation). However, the number of neurons is rather small as only 2.5% of all NURR1 immunoreactive cells present in these groups were BrdU labeled (Fig. 7D).

### Apoptosis

**Figure 8:**
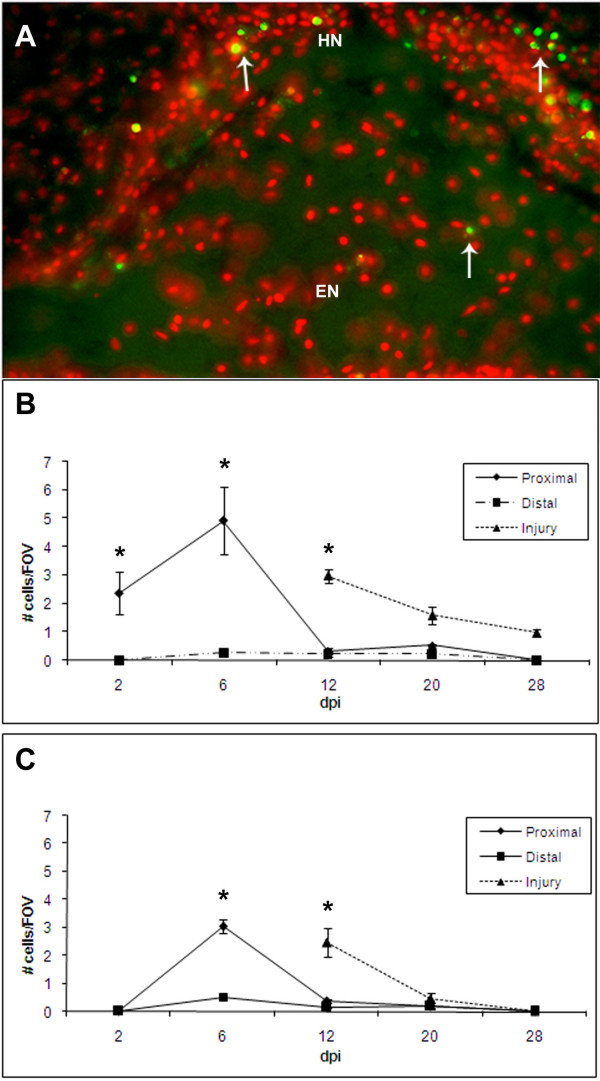
**Apoptosis during radial nerve cord regeneration**. (A.) Longitudinal section of regenerating RNC at 6 dpi showing TUNEL labeling (green) in cells undergoing apoptosis (arrows) (counter-stained with Hoescht-red). Apoptosis occurs in both (B) hyponeural and (C) ectoneural components of the regenerating radial nerve cord peaking at day 6 dpi in the proximal nerve stumps and at 12 dpi in the regenerating cord that is forming at the injury site. Little or no apoptosis occurs in areas distal to the injury. Regenerated tissues in the injured area do not appear until 12-dpe. Each point represents the mean ± S.E. of five animals. When compared to distal, significant differences are observed at various stages in the proximal and injured areas *p < .05, **p < .01. HN – hyponeural component, EN – ectoneural component. Bar = 25 μm.

To determine the cellular events that occurred during RNC regeneration following a transection-type injury, we decided to examine if programmed cell death was occurring (Fig. 8). As soon as 2 dpi, apoptotic cells were present in the HN component of the proximal stumps. The highest number of apoptotic cells in the proximal stumps occurred at 6 dpi, in both the HN and EC bands, although the magnitude of apoptotic cells was higher in the HN band. By 12 dpi and on to 28 dpi, few if any apoptotic cells were observed in the nerve proximal to the injury site. In contrast, at 12 dpi, a large wave of apoptosis occurred in the regenerated RNC that had just formed over the injury gap. The number of apoptotic cells in the regenerating RNC dropped in the two subsequent stages, until at 28 dpi, no apoptosis was observed in the EN and only in 1% of cells of the HN band.

### Spherule-containing cells

Two types of spherule-containing cells have been described in our system: morulas, which are highly basophilic and stain dark purple with toluidine blue, and spherulocytes, which are recognized by a monoclonal antibody prepared against sea cucumber tissues [28,32]. Only about 5% of spherule-containing cells are recognized by both markers.

**Figure 9:**
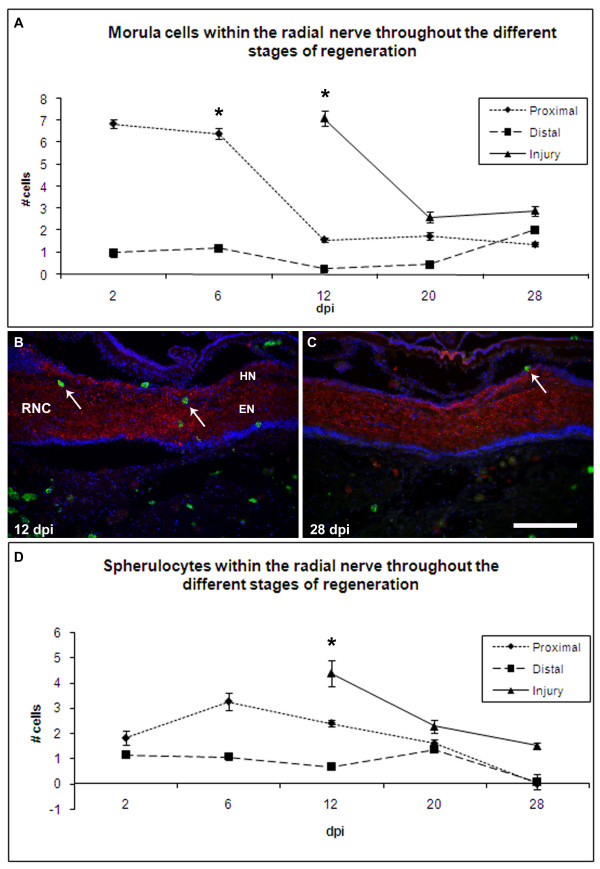
**Changes in spherule-containing cells during radial nerve cord regeneration**. The number of morula cells and spherulocytes increases in the regenerating radial nerve cord following transection. Morulas were detected using Toluidene blue (see Fig. 1). Quantitative analysis show that (A) the number of morulas peak between 2 and 6 dpi in the proximal nerve stumps and at 12 dpi in the regenerating cord that is forming at the injury site. Few changes are observed distal to the injury site. (B-C) Spherulocytes (green, arrows) were detected using the Sph2 antibody, a monoclonal antibody that recognizes the spherulocyte population. Longitudinal sections of animals at (B) 12 and (C) 28 dpi show the presence of spherulocytes within the nerve cord in early but not in late regenerating stages. Sections were also labeled with anti-galanin (red) and Hoeschst nuclear dye (blue). X's denote the injury site (D) Quantitative analysis show that the number of spherulocytes peaks at 6 dpi in the proximal stumps and at 12 dpi at the injury site, with little change at distal sites. Regenerated tissues in the injured area do not appear until 12-dpe. Each point represents the mean ± S.E. of five animals. When compared to distal, significant differences are observed at various stages in the proximal and injured areas *p < .05, **p < .01.

Few (less than1 per visual field), if any, morula cells were found within the normal RNCs (see Fig. 1). (As mentioned earlier, fields of view, FOV, were obtained with the 40× objective and encompassed an equivalent area in all animals). Changes in their number within the RNC were already seen at 2 dpi, when they invaded the nerve stumps. They achieved their highest numbers at this stage, when the average number of cells per field of view within the proximal stumps was 6.9 + 0.2 cells per FOV (Fig. 9A, and see Fig. 1). Most cells were closely apposed to the CT band that separates the HN and EN components; this is the same location where they are normally found in an uninjured nerve. However, as a response to the injury, morulas were also found interspersed within the neuropile, more commonly where the nerve stumps were engorged, or fan-shaped. By 6 dpi, the number of cells in the proximal stump remained the same (6.4 + 0.24 cells per FOV) as in the previous stage. However, some of these cells were also associated with the RNC cell bodies, in both the HN and EN, adjacent to the coelomic side and also with the regrowing fibers that originated from the nerve stumps. At 12 dpi the number of morulas in the proximal RNC decreased to levels just slightly higher (1.6 + 0.1 cells per FOV) than the basal level and remained at this level until 28 dpi. The changes in the morula population only occurred proximally to the injury site since distal to the injury site the cell numbers were not significantly different from uninjured nerves at any stage studied. The number of cells was much higher in the new RNC bridging the injury site at 12 dpi, on average there were 7.12 ± 0.35 cells per FOV. At 20 dpi, the average number of cells in the regenerated RNC declined to 2.61 ± 0.25 cells per FOV; similar to the number of cells found at 28 dpi (2.89 ± 0.23 cells per FOV)(see Fig. 1).

The number of spherulocytes within the RNCs also underwent changes during regeneration (Fig. 9D). These cells were not normally found in uninjured nerves, however, as soon as 2 dpi they were found within the proximal stumps (1.8 ± 0.27 cells per FOV); and to a lesser extent distal to the injury (1.1 ± 0. 07 cell per FOV). A consecutive increase was seen in the RNC stump by 6 dpi, when on average 3.23 ± 0.34 cells per FOV were observed in the RNC. By 12 dpi, the number of spherulocytes proximal to the regenerated RNC decreased, but still remained significantly higher than the basal level (see Fig. 9B). This was accompanied by a relatively high infiltration of these cells into the new RNC that had on average 4.34 ± 0.51 cells per FOV. The number of cells seen in the regenerated RNC by 20 dpi decreased to 2.24 ± 0.25 cells per FOV; the range of cells in proximal and distal parts relative to the injury site was 1.59 ± 0.17 to 1.34 ± 0.04 per FOV. Distally, the spherulocyte population remained unchanged from 3 dpi until 20 dpi at less than 1.4 cells per FOV. At 28 dpi, spherulocytes could only be detected in the regenerated portion of the RNC (see Fig. 9C), and although their levels were lower than those of previous stages, they were still significantly higher than in uninjured nerves.

## Discussion

### The regenerated RNC

Our results clearly show that the RNC of holothurians can regenerate within a month following a transection. The regenerated RNC looks amazingly similar to the non-injured RNC as judged by several histological and cellular parameters: It is well organized into ectoneural and hyponeural components, there is no scar or fibrotic tissue, the number of cells is similar to those found in uninjured tissue, neurons are localized to the nerve periphery, and spherule-containing cells that have infiltrated the injured cord have all but dissapeared. This is an amazing regeneration feat for an adult deuterostome, particularly if we consider that the RNC conforms the echinoderm CNS [24,29,30] and has been proposed to be evolutionary related to the vertebrate CNS [36-38].

Although we lack direct proof, a large amount of circumstantial evidence suggest that regeneration is not solely the joining back of the severed nerve stumps but that actually a new portion of the RNC is generated. First, from the moment it is cut, the wound forms a V-shaped structure where the nerve stumps are initially separated by a gap, and then by connective tissue. Second, most of the ongoing cellular division occurs within the regenerating RNC. Third, other tissues that surround the nerve and that also regenerate, do so by forming new tissues. This is particularly true of the surrounding muscle and connective tissues, which regenerate in parallel to the RNC and where very small differences in organization, cell density and texture distinguish the "new" tissues from the adjacent "old" [28]. Fourth, the initial and most visible regeneration event is the outgrowth of fibers from the RNC stumps. These outgrowths increase with time and eventually completely fill the space between the stumps. The latter retain their original morphology throughout regeneration, and it is the space in between that grows and becomes organized. Finally, recent work in another holothurian, *Eupentacta fraudatrix*, also shows regeneration of the RNC to be newly formed tissue [23].

In some ways, the formation of the new RNC can be compared to the "new" RNC segments that grow when the tip of the arm is severed in starfish or crinoids [19,20,22]. In all these cases the regenerated arm tip eventually grows to form new segments that include all the components found in the RNCs. Experimental evidence for the formation of a new RNC also comes from other closely related deuterostomes, the ascidians, that have an impressive ability to regenerate main components of their nervous system as has been shown by Bollner and colleagues [17,18] in studies where the main neural ganglion regenerates afer ablation. Similarly, lower vertebrates are able to regenerate a lost segment of the spinal cord as has been shown by nerve cord injury in amphibians [39,40] and fish [1].

### Phylogenetic analysis of regeneration following transection

How does the regeneration of the holothurian RNC compares to that shown by other animals? To answer this question we will focus on those experiments where injury to the CNS is done by transection of the main nerve cord (or spinal cord). This includes studies in protostomes, such as mollusks and annelids and lower vertebrates, such as fish and urodele amphibians. It also includes developing vertebrates, since experiments have shown that spinal cord regeneration can occur at particular developmental stages of some higher vertebrates, such as chicks or marsupials, and then becomes restricted as development continues [41,42].

RNC regeneration in *H. glaberrima *shows strong similarities to other animal species known to be capable of axonal regeneration and/or formation of a missing part of their CNS. In most species where CNS regeneration takes place, two events can be highlighed to occur: the outgrowth of fibers from the nerve stumps and the absence of fibrotic tissue or glial scar. The growth of fibers into the lessioned area provides the first indication of the regeneration process. As has been reported by TEM in the sea cucumber *E. fraudatrix *[23] and by immunohistochemistry in the starfish, *Asterias rubens *[22], these fibers originate from the lesioned stumps and move across the lesion eventually forming a bridge between the two nerve stumps. Similarly, in urodele larvae, fibers extending from each of the cut ends grow across the gap and eventually form a bridge connecting the two nerve ends [39]. New fibers are mainly attributed to the re-growth of fibers from lesioned cells as has been shown by retrograde labeling at the time of transection [43,44]. Another common observation in regenerating animals, including holothurians, is the fact that there seems to be a directionality to the process, since fibers extending from one stump are directed toward the opposite stump and are rarely observed to be traveling toward another target, suggesting the participation of soluble factors originating from the severed stumps and/or guidance molecules in the surrounding environment.

The absence of a glial scar or fibrotic tissue in regenerating cords is another characteristic of those animals capable of regenerating their main nervous system. No scar tissue is detected or described in studies showing regeneration in urodeles, fish or echinoderms. Similarly, chick embryos that are capable of regeneration do not form scar tissue [41]. In fact, what investigators regularly show to occur during the regenerative response is an increase in cells or cell activity associated with the removal of the injury debris and of apoptotic cells.

There are also differences in the regeneration process among animals. One of these is the level or extent at which regeneration occurs. In holothurians, the regenerated RNC appears almost indistinct from the non-injured RNC. However, in other organisms where it has been studied, the regenerated cord is not fully equivalent to the normal one in spite of fibers crossing the lesion site. For example, in the regenerating chick embryo, fibers do cross the transection site and functional regeneration occurs, but the site of transection shows a slight disorganization of the gray matter [41]. Another example is the regenerating spinal cord of bullfrogs that have been transected as tadpoles, where the transection site is still visible 8 months after injury (and 6 after metamorphosis), showing a considerable constriction in size [15]. The differences can be striking, as occurs in the lizard where it has been shown that the regenerated spinal cord in the tail is considerably different from the uninjured cord and that it only contains descending fiber tracts from the old cord and scattered glial cells [45]. Other species show subtle differences, as in the case of regenerating 1 year-old goldfish, where the regenerated spinal cord appeared normal after 90 days post injury, but there were no motor horn cells in the regenerated area [46]. In *H. glaberrima*, the regenerated RNC has NURR 1 and GFS expressing neurons (albeit in lower numbers) and appears to be normal according to all anatomical parameters used. Nonetheless, more detailed experiments are needed to fully determine that all cell populations and fiber connections are present.

Finally, it is important to acknowledge that structural differences do exist in the regenerating nervous systems of animals. The echinoderm ganglionated nerve cord differs greatly from the regenerating nerves of some invertebrates where only fibers are found, and from the complex cellular and fiber organization of the vertebrate spinal cord. Thus, it remains unclear whether similar events occur during the regeneration of the different structures. Nonetheless, it will be by performing comparative studies, such as this one, that the commonalities and differences in nervous system regeneration among animals will be established.

### Role of Cell division and apoptosis

Two related and at the same time, opposite processes are occuring within the RNC during regeneration; cell proliferation and cell death. The two show a spatial and temporal profile that seems perplexing because they seem to be occurring simultaneously; both show a peak in the nerve stumps at 6 dpi and a peak within the regenerating RNC at 12 dpi.

#### Apoptosis

In vertebrates, apoptosis is associated with neurons that have been injured at the time of transection, but also with a period of secondary injury where the lesion size increases due to inflammation, immune-related events and hemorrhage [47-49]. Therefore, in our system, apoptosis within the nerve stumps seems to occur in cells whose fibers were damaged by the transection and that eventually failed to survive, undergoing apoptosis during the first week following injury. This was also observed at the TEM level in regenerating RNC of *E. fraudatrix *[23] and in regenerating fish CNS where apoptotic cells are seen for a long period of time following the lesion [50].

A different explanation might be involved in explaining the increase in apoptosis observed within the regenerating RNC in the second week following the transection. In this case the apoptosis event might be similar to what has been shown to occur during embryonic development in vertebrates, where about half of the neurons that are formed in the spinal cord undergo apoptosis [51]. Neuronal death in regenerating spinal cord has also been reported in the tail of the fish *Sternachus *[1,52]. In these fishes, younger segments of the regenerated spinal cord have a larger number of neurons than older segments, once again suggesting that the regenerating spinal cord recapitulates the known embryonic developmental process of overproducing neurons that will eventually be discarded.

#### Cell division

Cell division has been shown to occur in the regenerating arms of several echinoderms, mainly crinoids and starfish [20-22]. In crinoids, the nerve stump is also an active place of cell proliferation, although the dividing cells in the early stages appear to be non-neuronal [20]. In the starfish *Asterias rubens*, it was found that cell division in the regenerating nerve began 6–7 days following transection and increased during the next 2 weeks, gradually decreasing to control levels by day 60 post-injury [22]. Similarly, our results show cell division to occur within the ectoneural and hyponeural components of the nerve stump, but even more within the regenerating RNC. However, it is still unclear what type(s) of cells are proliferating since support cells, macrophages or some type of precursor cell could be accountable for the extent of cell division. This is a crucial issue that will be addressed below. Nonetheless, in recent studies Mashanov and colleagues [23] describe neurons and glial cells undergoing division in the regenerating RNC of *E. fraudatrix*.

The overall pattern of cell division within the regenerating RNC has some similarities to what has been shown to occur in the adjacent muscle and connective tissue, where cell division peaks between 6 and 12 days following injury [28]. These two weeks of extensive cell proliferation are undoubtly responsible for the finding that, at 30 dpi, animals have the same number of cells in the regenerated and in the uninjured RNCs.

Formation of new neurons has been shown to be an important process in animals where CNS regeneration is known to occur. In fish, BrdU labeling together with back-tracing techniques have shown that in the adult fish, neurons are regenerated from proliferating precursors following CNS injury [53]. In urodeles, new neurons are known to originate from proliferating ependymal cells [14]. Whether new neurons take part in the regeneration of the spinal tract observed in the chick embryo and the neonateal opposum is not known [54]. However, recent results suggest that stages where the ability to regenerate is still present, coincide with those stages where there is ongoing formation of new neurons [55]. On the other hand it is important to emphasize that some vertebrates can undergo functional regeneration with little or no contribution of neurogenesis, or that new neurons can originate from undifferentiated precursors.

In view of these findings, our results showing that neurons are present within the regenerated RNC and that some originate from dividing precursors provide for interesting comparisons to other systems. At the same time we must reconcile the finding that the densities of GFS and NURR1 neurons are lower in the regenerated RNC. One possible explanation is that the appearance of neurons in the regenerated RNC is a late event and that at 62 dpi some neuronal precursors have not fully differentiated. This would be consistent with experiments in starfish, where the first neurons observed in the regenerated segment following transection of the RNC were not observed until 28 days following injury [22]. These results might appear to contradict the observations by Mashanov and colleagues [23] in *E. fraudatrix*, who observed some neuronal division in the early stages of RNC regeneration, however, this might be explained when we take into account that early division was observed using TEM, while we used markers for differentiated neurons. Nonetheless, it is important to emphasize that in both *E. fraudatrix *and *H. glaberrima *neurons appear in the regenerated RNCs and that at least some of these neurons originate from dividing precursors. Similarly, in crinoid regenerating arms, some BrdU labeled neurons were recognized using electron microscopy, suggesting that in this echinoderm, neurons also originate from dividing precursors [21]. However, there are striking differences between neuronal populations since the GFS neurons were not observed to express BrdU, suggesting that they do not originate from dividing precursors. Once again this is consistent with studies in the transected starfish RNC where none of the neurons identified with the S1 neuropeptide (the starfish equivalent of GFS) showed BrdU labeling, even when enough time was allowed following the BrdU treatment for differentiaton to occur [22].

In order to fully identify the origin of neurons it will be important to determine the role of glia in holothurian RNC regeneration. Particularly in view that in *E. fraudatrix *Mashanov and colleagues propose that glial cells might be paving the path for neuron and fiber migration and even differentiating into neurons [23]. The role of the glial cells is not the only question that remains to be studied; several other questions remain to be answered. For example, does the observed anatomical regeneration provides for complete functional recovery? In many other model systems the extent of regeneration has been evidenced by backtracing neuronal connections and comparing the regenerated versus the uninjured connections. However, in view that little is known about the echinoderm radial nerve circuitry and nervous system physiology, it is not possible to determine at present to what extent there is morphological connectivity. Similarly, in other animals functional recovery can be documented by behavioral tests, that, at present, are not available for holothurians.

### Comparison with other wound healing/regenerative processes

Transection of the nervous system, whether experimentally induced or by accidental cause rarely, if ever, exclusively affect the nervous system. Thus, it is always important to view the regeneration of the nervous component as a process that occurs parallel to the regeneration of surrounding tissues. Granted, there are nerve-specific processes, such as axonal regeneration and synaptic specificity, that only occur in the nervous system. However, it is interesting that events that occur in the surrounding muscle and connective tissue previously published by our group [28] parallel those occuring within the nerve cord itself.  One of them has already been addressed above: the pattern of cell division, showing that in muscle, coelomic epithelium and connective tissue the proliferation pattern coincides with that observed in the regenerating RNC.

Another event that provides for an interesting comparison is the presence of morula or spheruloytes cells within the RNC. These cells are rarely observed in uninjured nerves, however, their numbers increase after injury. This increase in cell numbers parallels what occurs within the regenerating intestine [32] and following the transection injury in the adjacent tissues [28]. These cells have been proposed to play important roles in extracellular matrix remodeling, phagocytosis and antimicrobial activity, among others [32]. The presence of these cells within the regenerating nerve is quite intriguing and should be studied in further detail.

The extent of apoptosis observed after nerve transection can also be compared to what occurs during intestinal regeneration. We have recently completed a study of apoptosis during intestinal regeneration showing that high levels of apoptosis take place in the mesothelium 7 days following evisceration (unp. Observation). In both events, nerve regeneration and intestinal regeneration, apoptosis is rarely observed in the early days (2–5 days) following the injury or evisceration but occurs more likely during the second week (7–14 days). Moreover, apoptosis is more likely to take place within "new" tissues that have regenerated such as the newly formed intestinal blastema-like structure and the regenerated RNC, than in adjacent "old" tissues, such as the remaining nerve stumps or the intestinal mesentery.

For many investigators involved in studies of neurogenesis or neuronal regeneration, their focus has been strictly on the nervous system. However, our previous results show that the same events that occur during wound healing, also occur during intestinal regeneration [28]. And now we show that some of these events also occur during nervous system regeneration. Moreover, at the species level, the same species that can successfully regenerate their nervous systems are known to be exceptional regenerators of others tissues or organs, not just the nervous system. Thus, a major implication of our work is that the capacity to regenerate nervous system might be determined by the same processes and mechanisms in all tissues and that some animals have developed or retained these capabilities while in others they are absent. Although there are obvious exemptions to this statement, such as liver regeneration in vertebrates, the general rule remains that animals that have some capacity to regenerate their nervous system are, in general, good regenerators of many tissues or organs. If this is correct, then investigators interested in deciphering the mysteries of nervous system regeneration should also focus on regenerative or wound healing phenomena in other tissues and organs and on species where these regenerative processes are prominent.

## Authors' contributions

JSMR carried out most of the experiments and helped in the analyses of the data and preparation of the manuscript. ARMS performed the birthdating experiments, analysed the data, and prepared the corresponding figures. JEGA conceived the study, participated in its design, data analyses and helped in the manuscript preparation. All authors read and approved the final manuscript.
